# Coping with dysmenorrhea: a qualitative analysis of period pain management among students who menstruate

**DOI:** 10.1186/s12905-022-01988-4

**Published:** 2022-10-05

**Authors:** Fódhla Ní Chéileachair, Brian E. McGuire, Hannah Durand

**Affiliations:** 1 School of Psychology, University of Galway, Galway, Ireland; 2 Centre for Pain Research, University of Galway, Galway, Ireland; 3 Division of Psychology, Faculty of Natural Sciences, University of Stirling, Stirling, Scotland, UK

**Keywords:** Dysmenorrhea, Period pain, Qualitative analysis, Students

## Abstract

**Background:**

Dysmenorrhea, or period pain, affects up to 95% of menstruating individuals and is a common cause of educational absenteeism among students who menstruate worldwide. Evidence suggests that students may lack sufficient knowledge about their menstrual health, which may impede self-management. The aim of the current study was to explore pain management strategies used by students in Ireland with painful periods and to identify their unaddressed needs across physical, psychological, educational, and social domains.

**Methods:**

This study used a qualitative, interpretive design and opportunity sampling approach to collect and interpret individual accounts of dysmenorrhea from third-level students in Ireland. Data from 21 students were collected using semi-structured online one-to-one interviews and analysed using reflexive thematic analysis.

**Results:**

Analysis resulted in the construction of five themes: (1) *Pain management is self-directed trial-and-error,* (2) *Home as safe haven,* (3) *Prioritising productivity over pain,* (4) *We’re missing an option between ‘normalise’ and ‘medicalise’, and* (5) *Cycle of censorship and concealment.* Overall, limited formal education on dysmenorrhea and prevailing negative attitudes towards menstruation create an unsupportive environment for students to learn adequate coping skills. Beyond education, menstrual stigma may also restrict the availability of clear management guidance in domestic and medical spheres. Experiences of dysmenorrhea were also influenced by the COVID-19 pandemic, where work-from-home measures were viewed favourably by individuals with dysmenorrhea.

**Conclusions:**

This study indicates that students in Ireland are inadequately prepared to cope with dysmenorrhea. The current findings have substantial implications for evaluating and reforming current menstrual education standards, in addition to clarifying the negative effects of social stigma on menstrual health literacy.

**Supplementary Information:**

The online version contains supplementary material available at 10.1186/s12905-022-01988-4.

## Introduction

Dysmenorrhea, known as ‘period pain,’ refers to pain experienced just before or during menstruation [1, 2]. It may affect up to 95% of individuals who menstruate [3, 4] and is prevalent across brackets of socioeconomic status, ethnicity, and nationality [5]. Dysmenorrhea can also occur secondary to several gynaecological disorders, commonly endometriosis, fibroids, or adenomyosis. That said, dysmenorrhea itself can cause debilitating physical symptoms, including severe pelvic and/or abdominal pain, vomiting and fainting, in addition to psychological symptoms, like dysphoria and anxiety [6, 7]. Despite the prevalence and impact of dysmenorrhea, there is growing concern that from the age of menarche, the first occurrence of menstruation, individuals who menstruate receive limited education on managing dysmenorrhea [8, 9].

Insufficient information on menstrual health proves a challenge to menstrual health literacy, defined as the capacity to make decisions based on acquiring, understanding, and processing menstrual health information [9]. Evidence from Australia [8], Spain [6], and the UK [10] highlights that young individuals demonstrate gaps in their knowledge of menstrual health. Among secondary school students in the UK, Randhawa and colleagues [10] reported that less than 10% of participants could accurately define endometriosis and, moreover, that approximately 30% could not identify whether their period was regular. These gaps in menstrual health literacy have been linked to misinformation across a number of aspects of pain management, like the normalisation of severe pain and low help-seeking rates among individuals with dysmenorrhea [1, 6].

There are concerns that some cases of dysmenorrhea may be symptomatic of undiagnosed conditions, like bleeding disorders [11] and endometriosis [12] and may be erroneously classified as typical of menstruation by healthcare practitioners or individuals themselves [13]. Moreover, popular over-the-counter (OTC) analgesics may not provide effective pain relief [2, 14]. OTC analgesics vary widely in terms of their efficacy for period pain relief, with current guidance recommending the use of and non-steroidal anti-inflammatory drugs (NSAIDs) [14]. While some effective NSAIDs are available OTC, current evidence suggests that OTC painkillers with the lowest effectiveness for period pain relief are among the most commonly used by individuals with dysmenorrhea [8, 14], as exemplified by the popular use of paracetamol by Irish students [15]. Further, while hormonal contraception or prescription-strength NSAIDs are cited as highly effective methods of regulating dysmenorrhea, knowledge of these options may be limited, as they are only available through medical consultation in Ireland [16].

Recent publications suggest that most individuals who menstruate do not seek medical advice for severe period pain [15, 17] and may be unaware of potential health conditions or effective options to minimise symptoms [5, 10]. Help-seeking, which is broadly defined as proactively searching for guidance to address problems [18], was observed among only 11% of individuals with dysmenorrhea across sixteen countries [8]. A reliance on potentially ineffective relief methods among students is a concern, as research indicates that dysmenorrhea is a common cause of educational absenteeism and presenteeism, in which symptoms interfere with attendance and/or participation [7, 19]. In this manner, students with dysmenorrhea may contend with unmet needs across facets of health and wellbeing, as well as facing difficulties in their educational development.

Help-seeking behaviours, whether through friends and family or through formal healthcare consultations, are notably complex and may be influenced by low health literacy and psychosocial factors, like embarrassment, stigma, and perceptions that menstrual pain is not a legitimate health concern [1, 8]. Recent studies have documented concerns among women that the legitimacy of dysmenorrhea may be doubted by healthcare practitioners [20], where individuals believe some doctors do not take their symptoms seriously, and by individuals with dysmenorrhea themselves, who may normalise severe symptoms [1, 6, 20]. Moreover, experiences of dysmenorrhea are heavily influenced by sociocultural attitudes [21], where periods may be regarded as shameful in some sociocultural contexts. In 1996, Kissling [22] highlighted that young women in the US receive mixed-messages about menstruation, which is framed as a sign of maturity but ultimately as an undesirable part of life for the individual and their social surroundings. Recent publications have drawn similar conclusions, where social attitudes towards menstruation may perpetuate a confusing perspective that menstruation is simultaneously natural and abhorrent [23]. Management practices for menstrual pain may not simply be based on the experience of dysmenorrhea, but influenced by a blend of psychosocial factors, such as the surrounding social environment, legitimacy, and limitations in menstrual health literacy. As a blend of psychological, social, and educational factors may account for disparities between symptom severity and coping responses, exploring the perceptions and experiences of university students with dysmenorrhea using a qualitative approach may clarify perceived barriers to improving knowledge and care of menstrual pain.

### Perceptions of dysmenorrhea

The common sense model (CSM) of self-regulation [24, 25] comprises a useful framework through which to consider personal representations of dysmenorrhea and relevant coping responses, such as low rates of help-seeking. The CSM proposes that individuals construct perceptions of their condition, comprising cognitive and emotional representations from the following domains: (1) identity *(e.g., symptoms)*; (2) timeline; (3) consequences *(e.g., pain, absenteeism)*; (4) perceived causes *(e.g., perceptions of symptom normality)*; (5) control beliefs *(e.g., use and effectiveness of pain-relief medication)*; (6) coherence *(e.g., menstrual health literacy)*; and (7) emotional responses *(e.g., feelings of negativity).* In their study on the influence of dysmenorrhea beliefs on behavioural responses, Chen and colleagues [20] reported that most respondents characterised dysmenorrhea as a ‘normal’ aspect of womanhood. Individuals with dysmenorrhea may lack awareness and knowledge of menstrual normality, which may impede help-seeking and effective pain management, as dysmenorrhea may not be identified as a legitimate condition [6, 8, 20]. Moreover, individuals may only have access to a limited and potentially skewed pool of information, based on their own experiences and/or similar experiences of family members, with which to construct representations of a ‘normal’ period [10, 13]. Constructing accurate representations of dysmenorrhea may be particularly challenging for younger groups, like students, who may not have access to evidence-based sources of menstrual health information from menarche [10, 21].

Despite the prevalence of dysmenorrhea worldwide, recent evidence suggests that the majority of individuals with dysmenorrhea may rely on ineffective techniques to manage pain [8]. Potential barriers, such as low menstrual health literacy and the normalisation of pain-related disturbances, that may impede access to effective management supports are a growing concern, particularly as the perceived consequences of dysmenorrhea notably traverse physical, psychological, and social health facets. In the student population, as inadequate symptom relief distinctly interferes with attendance and participation, there is an evident need to investigate potential barriers impeding effective coping. Moreover, given the influence of the social context on perceptions of menstruation, there is clear scope to explore unmet needs of the student population with dysmenorrhea. A qualitative exploration of experiences with dysmenorrhea across these interconnected facets of health may aid interpretations of potential inconsistencies between documented severity of dysmenorrhea and individuals’ responses to the condition. Specifically, the current study has two primary aims: (i) to explore the strategies and coping behaviours for self-management among students; and (ii) to identify internal and external factors that may contribute to ineffective management strategies for dysmenorrhea.

## Research questions

1. What strategies do Irish university students with dysmenorrhea adopt to cope with physical, psychological, educational, and social consequences of dysmenorrhea?
2. What factors inhibit and/or facilitate effective management of dysmenorrhea among Irish university students?

## Method

### Research design

Using a qualitative design, the present study adopted a phenomenological approach to describe the subjective first-hand student experiences of dysmenorrhea. Phenomenology provides a means of targeting specific phenomenon, such as menstrual pain, and describing its core aspects using individual accounts [26, 27]. Data related to experiences of dysmenorrhea were collected through semi-structured interviews with individual participants. Reflexive thematic analysis (TA), as described by Braun and Clarke [28, 29] was used, as it enabled the development of nuanced accounts of the target phenomenon, in line with the principles underlying phenomenology [27]. Reflexivity refers to the practice of evaluating and monitoring the beliefs, values, and perspectives of the researcher during the analytic process [29], ensuring a maintained awareness of coding and theme construction choices. A reflexive ‘log’ was recorded to note thoughts and observations during the analysis to remain mindful of any internal assumptions and/or biases on the subject matter. In line with current standards of qualitative description, the Consolidated Criteria for Reporting Qualitative Research (COREQ) [30] and the evaluation tool for reporting TA [31] guided the present report. A COREQ checklist is provided in Additional file 1.

### Participants

Third-level students (≥ 18 years of age) registered at universities on the island of Ireland who previously and/or currently experienced dysmenorrhea were eligible to participate. Given the focus on primary dysmenorrhea (i.e., pain experienced during menstruation in the absence of an underlying gynaecological cause), individuals with any known diagnosis of a gynaecological condition that may incur secondary dysmenorrhea (e.g., endometriosis) were not eligible for inclusion. Individuals with dysmenorrhea where period pain could potentially be attributed to an underlying condition but was not definitively diagnosed (i.e., undifferentiated dysmenorrhea) were eligible to take part. Participants (*N* = 21) self-selected to engage during March–May 2021. Participants were recruited using advertisements on social media, as well as through student societies and organisations in Ireland relating to health, period poverty, and social issues. Individuals expressing interest were invited to answer a brief demographic questionnaire using Qualtrics® software (*v. 2021)* to indicate consent to partake, and to provide their university email address to schedule their interview. A total of 21 participants took part, spanning undergraduate to doctorate levels of education (see Table 1). Most students (*n* = 20) attended universities in the Republic of Ireland and one participant was registered at a university in Northern Ireland. Notably, the majority of participants had previously sought help from medical professionals (*n* = 17) to manage dysmenorrhea.

**Table 1:** Demographic characteristics of participants

Participant characteristics	*N*
	21
*Gender identity*	
Women	20
Non-binary	1
*Age (in range)*	
18–29 years	17
30–39 years	3
40–49 years	1
*Educational level*	
Undergraduate	13
Master	4
Doctoral	4
*Nationality*	
Republic of Ireland	16
Other^a^	5
*Experience of help-seeking*	
Yes	17
No	4

^a^Other participants were from India, Italy, Malaysia, Northern Ireland, and the United States (all n = 1)

### Data collection

A semi-structured interview guide (Additional file 2) was constructed to explore experiences of dysmenorrhea and subsequent health behaviours. The guide was developed in line with Kallio and colleagues [32] by retrieving and compiling literature and developing a comprehensive overview of topics *(e.g., menstrual health literacy, consequences of pain).* Following a pilot interview (*n* = 1), wording was adjusted to ensure questions were clear and open-ended. Pilot data were subsequently included in the overall dataset, as this individual fulfilled each inclusion criterion and consented to contribute their data. All interviews lasted approximately twenty minutes and were conducted over Microsoft Teams®, where participants were invited to remain on- or off-camera, as desired. Interview audio only was recorded using Microsoft Teams® or Audacity® *version 2.4.2* (2021), if an individual preferred to remain on-camera. Audio transcription was then completed, where 62% of files were transcribed using the ‘Dictation’ function in Microsoft Word® and edited manually. The remaining 38% were transcribed using Happy Scribe® (2021) software and reviewed thoroughly alongside audio to ensure accuracy.

### Data analysis

Reflexive TA, as outlined by Braun and Clarke [28, 29, 31] was used to construct and describe representational themes of data. TA comprises six phases: (1) data familiarisation; (2) initial code generation; (3) searching for themes; (4) reviewing themes; (5) defining themes; and (6) preparation of the written report [28]. Coding comprised a blended approach of both inductive and deductive approaches. Inductive coding entailed an iterative process of establishing commonalities within the initial data, coding these commonalities, and refining these base codes by progressing through the complete dataset. Coding was also guided by the deductive identification of components within the CSM [25], to ground the process within the framework of illness self-management. Following initial coding of the dataset, codes were reviewed and refined following interactive discussions between authors, where 10% of the data was also coded by the last author for critical examination. NVivo® *version 12* software was used to aid the coding process from phase two onwards.

## Results

**Fig. 1:**
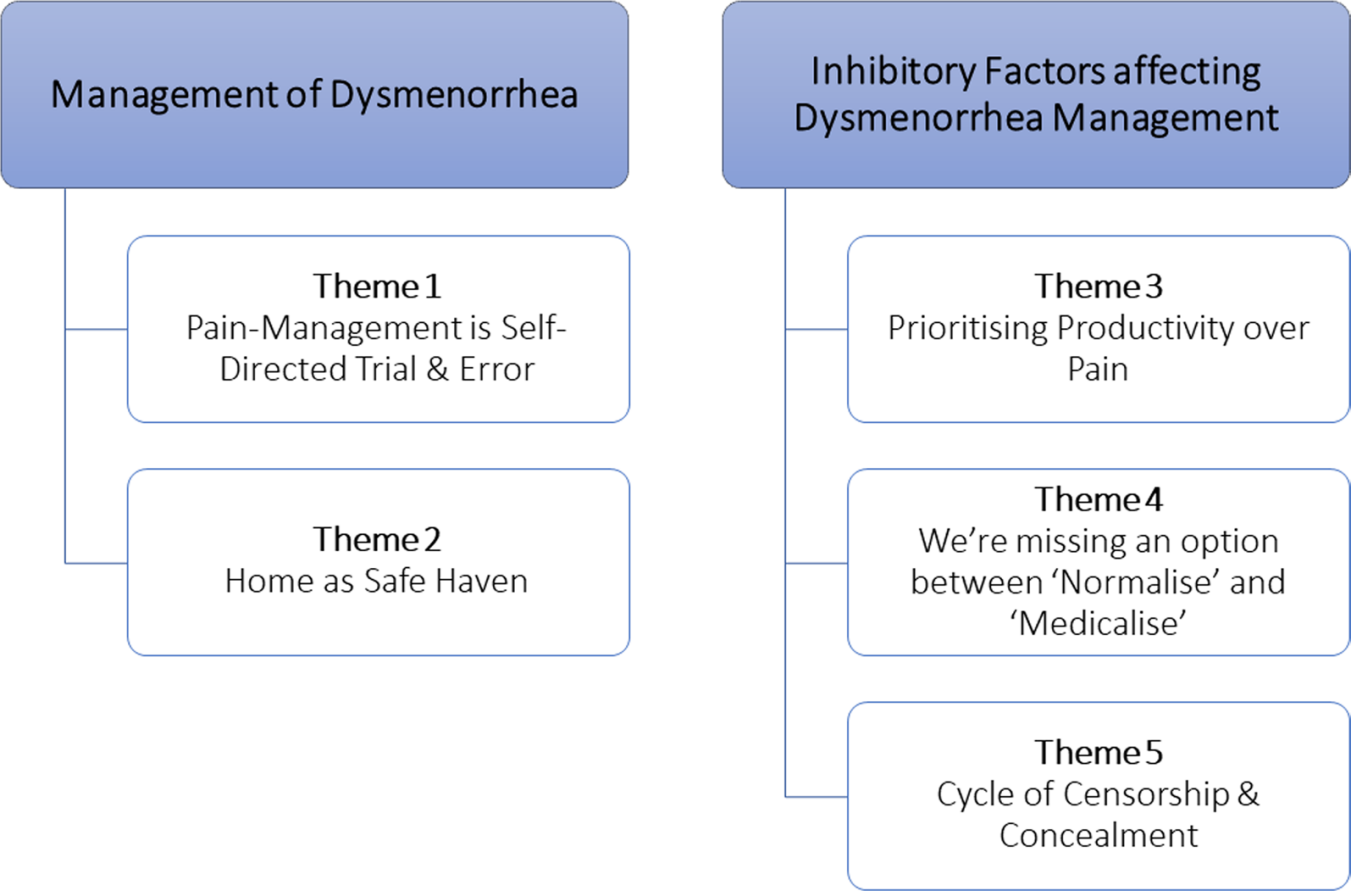
Visual depiction of finalised themes

A number of themes for each research question were constructed using reflexive TA [28, 31]. An overview of the shared experiences of dysmenorrhea is summarised below with respect to symptoms and coping mechanisms. Major themes are then outlined (see Fig. 1) and illustrated using quotations (see Tables 2, 3).

**Table 2:** Description of Themes 1 & 2

Theme	Quotes
Pain management is self-directed trial-and-error	“It was very much a ‘mechanical’ conversation, so none of it was about … pain management or what to expect.’ (P2) “You don't really learn much about it, apart from actual biological basis and even like PMS and stuff like the week before, like headaches, mood swings, like, that's really hard to cope with.” (P14) “If I take painkillers 'just in time' ….it stops the kind of onset of the pain, whereas if I leave the pain to settle in at all, the painkillers don't really … They're not really as effective.” (P16)
Home as safe haven	“You kind of want comfort knowing that there's hot water at home, there's you know like a shower you can sit inside or a bath with hot water and…” (P1) “Getting home would have been a big part of managing it…you just want to be in your familiar surroundings, like when you're that sore, and when you're in pain.” (P16) “It's been kind of the silver lining of like lockdowns and having to work from home and study from home … when you when you do get your period now, it's like you can sit there on a lecture and have your hot water bottle and… be comfortable.” (P9)

**Table 3:** Description of Themes 3–5

Theme	Quotes
Prioritising productivity over pain	“If I were to try to go to … a professor and talk about why I couldn’t come in or why I hadn’t gotten something done, I would claim some other form of sickness that is more **universally valid.**” (P7) “I wouldn’t want to be perceived as weaker than other members of the class.” (P20)
We’re missing an option between ‘normalise’ and ‘medicalise’	“I do wish that they would have spoken to me about the effect it would have on my menstrual cycle because I feel like I don't really know … if it's normal or if it's not normal.” (P15)
Cycle of censorship and concealment	“When you're, like, 11 and you get that … sex talk in one room and the boys are in another and they talk to the girls about periods, when they teach the boys about like wet dreams and, like, erections… I feel like we should all have to learn about all those.” (P4) “There was very little… information about periods themselves or how to deal with them. It was just… ‘sometimes you bleed, sometimes you don't, and then you use period products to clean that up,’ … very much a kind of dirty, taboo topic rather than something we should be open about, especially in an all-girls school.” (P21)

### Overview of experiences with dysmenorrhea

‘Period pain’ was described as abdominal pain during menstruation. Looking to the ‘identity’ domain of the CSM [24], descriptions of pain were diverse, featuring a continuous, dull, muscular ache, intermittent sharp cramps, and/or the sensation of organs twisting. Ratings of severity varied, ranging from moderately sore to debilitating. In particular, two participants recalled instances where pain was so severe, they sought emergency medical attention in emergency departments. Participants highlighted regularly experiencing pain and discomfort, like back pain, headaches, vomiting, and the sensation of muscle weakness during their period. Dysmenorrhea was reported to negatively affect students, from poorer concentration and attendance at lectures, lower working capacity, and limited social engagement overall. The majority of participants (81%) had consulted a doctor about their condition, which is relatively high [1, 8]. Common coping behaviours included heat application, OTC analgesics, engaging in acupuncture and light exercise, in addition to taking prescription-strength painkillers and using hormonal contraception.

### Research question 1: coping strategies for dysmenorrhea

Two themes were constructed to capture features of management strategies amongst students with dysmenorrhea: (1) Pain management is self-directed trial-and-error; and (2) Home as safe haven.

#### Theme 1: pain management is self-directed trial and error

Most of it was trial-and-error… I would say I just had to figure it out myself. (P2)

This theme characterises the approach of finding effective mechanisms to manage symptoms of dysmenorrhea. In line with perceptions of controllability, the identification of strategies was framed as a gradual process of uncovering an individual routine to combat symptoms. Efforts to find suitable strategies were defined as ‘hit or miss,’ where attempts might lead to moderate relief or no relief at all. This process of ‘trial-and-error’ led some participants to design their own routine over time and gradually construct strong beliefs of symptom control, such as having hot water bottles readily available at work/home. Developing a knowledge base of effective treatments was gradual, where experience facilitated greater coherence and awareness of relief strategies. While it was possible for participants to develop their own pain-management routines over time, some reflected that additional help early on to identify these effective strategies would have been helpful.

What I honestly think would have been nice is if, when I had been younger and first experiencing these symptoms, someone could have told me what I learned through experience over the years… ‘you need a lot of rest, you need a lot of hydration…’ (P7)

Greater knowledge of relief options at menarche facilitated the trial-and-error process and was established within ‘open’ homes, where participants were guided by their parents. Supportive relationships with mothers helped in accessing information, particularly where their mothers’ experiences of painful periods were similar to their own. Other participants reflected on their limited knowledge of pain relief at menarche, noting that they felt uncomfortable about approaching their parents with menstrual concerns. Moreover, differing cultural responses to menstruation were identified as impediments to open family communication on the topic of period pain by both Irish national and international students. Lacking guidance from home, some participants were left to begin the process of ‘trial-and-error’ from scratch.

I just dealt with it myself and, I remember there was no Google and I didn’t have books for myself, so I went to the library…I used to talk to my mum but she felt shy. (P17)

Participants from Ireland reported receiving limited guidance during formal education. During biology classes on menstruation, concrete guidance for managing period pain was not discussed nor did Irish participants recall being directed to evidence-based sources of information. To develop a management routine for dysmenorrhea, participants with limited support generally turned to books or the internet for initial guidance, though they expressed that relying on the internet was hazardous due to inconsistent fact-checking practices.

#### Theme 2: home as safe haven

Getting home would have been a big part of managing it, because…you just want to be in your familiar surroundings when you’re that sore. (P16)

This theme describes a clear preference among students for coping with period pain at home. Home was viewed as the favoured environment to manage dysmenorrhea, as participants expressed beliefs that they were capable of controlling symptoms in their own abode. A sense of physical convenience was attributed to managing dysmenorrhea at home, due to the availability of hot water, comfortable clothing, and their own bed. Participants indicated that pain relief was often best achieved from resting in bed during the first day of pain, where they believed they were capable of completing work-related or university tasks while managing symptoms. Preferences for managing dysmenorrhea at home resulted in comparisons between home and expectations of the professional world. When periods occurred during the working week, participants faced the decision between remaining at home or facing their symptoms among peers in university and/or the workplace, where they believed their ability to control symptoms was restricted. A concern for participants was the lack of social acknowledgement of period pain in professional spheres. As such, participants reported using coping strategies for dysmenorrhea in university or at work, such as taking OTC analgesics, but further described efforts to conceal their symptoms from classmates to ensure no one was made to feel ‘uncomfortable.’

Home was portrayed as an acceptable environment to proactively manage symptoms without pressures of socially-determined ‘menstrual etiquette.’ The work-from-home policies employed during the COVID-19 pandemic from March 2020 were hailed as one positive outcome for students with dysmenorrhea, as this enabled them to engage in their home-based management strategies while attending online lectures, without seeking external permission to remain at home or having to take official leave. Considering the social context of menstruation, the privacy of home may aid students in coping with social implications of menstrual pain.

This is my first year of college, so it's all been online. So even if I'm in pain, I can still have the lecture on in the background. (P21)

### Research question 2: inhibitory factors

Three themes were developed to describe common aspects that impede effective management of dysmenorrhea among students: (3) Prioritising productivity over pain; (4) We’re missing an option between ‘normalise’ and ‘medicalise’; and (5) Cycle of censorship and concealment.

#### Theme 3: prioritising productivity over pain

As long as I was able to take my pain medication and go home and… get in bed with a hot water bottle… I think I’d still be able to do my work. (P9)

This theme describes the efforts of individuals with dysmenorrhea to give precedence to remaining productive at work and/or in university when experiencing pain. Striving to achieve usual standards was emphasised, where participants specified measures taken to ensure they could be productive. Within the narrative of ‘prioritising productivity’ in work and university, the question of period pain legitimacy was raised by participants. Though a common consequence of dysmenorrhea, participants expressed doubt that period pain would be considered a valid reason to be unproductive. Hesitancy to seek time off from work or lectures due to dysmenorrhea was evident, with participants preferring to complete work at home without seeking formal leave. Participants were inclined to endure their symptoms at work or feign a less stigmatised illness to avoid revealing the cause of their pain. Internal sources of pressure to remain productive were described, such as feeling guilty for missing a day’s work, in addition to external sources, such as the perceptions of employers and classmates. Taking time off to manage period pain was equated to being seen as an undesirable candidate for employers, with participants expressing fear that period pain may disincentivise employers from hiring them. As such, students indicated that their experience of dysmenorrhea in university or work may be framed as reflecting the ‘weakness’ of the individual rather than the severity of their pain.

You don’t want people in the workplace treated differently. If you say a period is a problem, then they won’t hire you. (P15)

#### Theme 4: we’re missing an option between ‘normalise’ and ‘medicalise’

There was a certain element of ‘this is just part of life, just get on with it.’ (P16)

This theme represents a perceived gap in options available to individuals with dysmenorrhea, where participants are generally given one of two ‘solutions’; to learn to tolerate their pain or to manipulate their menstrual cycle using hormonal contraception. A sense of normalising menstrual pain and related disturbances was emphasised by participants, who noted that while some doctors were sympathetic to their case, their experience was frequently labelled as ‘just unlucky.’ Participants expressed frustration with this view, particularly where their experience was often prefixed by the qualifier *‘just’* by medical professionals, functioning to minimise the severity of symptoms.

Someone told me I could suffer with endometriosis, but that I don’t ‘check all the boxes.’ So, because I ‘only’ have a painful period… it’s probably **just** that I’m unlucky, right? (P8)

Managing dysmenorrhea through hormonal methods also produced mixed views from participants relating to their control beliefs. Some hailed the relief they received from contraception, while others were perturbed by undesired side-effects. A further source of dissatisfaction with hormonal contraception was that it may also mask the characteristics of a period and preclude individuals from learning about their cycle. Others felt uncomfortable with their doctor’s recommendation of using contraception for period pain, noting that the goals of prescribing contraception may not align with their own. Specifically, participants expressed their view that doctors ‘medicalise’ menstruation by prioritising potential elimination of a monthly period altogether using contraception, rather than providing patients with pain relief guidance. Generally, while dysmenorrhea is uncomfortable, the view that dysmenorrhea does not represent the complete experience of menstruation was voiced, where menstruation itself can be a helpful sign of health and vitality.

I think it’s a pity that women are… told, like, there’s something wrong with their bodies …to me, it’s kind of a reminder of like ‘Ok, everything is normal and healthy in my body,’ (P9)

#### Theme 5: cycle of cultural censorship and concealment

You don’t talk about it, you don’t complain…you don’t bring it up with anybody… it’s… a secret kind of shameful thing for most of society. (P2)

This final theme represents the detrimental effects of social and cultural censorship of menstruation on effective dysmenorrhea management. Attempts to censor aspects of menstruation featured in interpersonal conversations, education, and media discourse amongst Irish national and international students. Culture was discussed in line with censorship, where both Irish and non-Irish participants felt that some cultural backgrounds placed greater emphasis on certain concealment practices. Advertisements for period products were mentioned, where they were perceived to be limited to aspects of cleanliness only and perpetuating connotations of impurity associated with periods.

I remember seeing advertisements for sanitary towels and tampons on TV when I was a kid and they always used blue liquid instead of red… when it's on TV, even when it's like publicly being talked about… it's still kind of a secret. (P2)

Participants from Ireland reflected in disbelief on the recent backlash against a Tampax® advertisement entitled ‘*Tampons and Tea’* in 2020, where the Advertising Standards Authority for Ireland received 84 complaints from members of the public lodged under four categories: general offence, demeaning, sexual innuendo, and suitability for children [33]. The advertisement, which aimed to instruct individuals how to insert Tampax tampons correctly, was subsequently removed from public television.

I was really surprised with the Tampax last year - The *‘get ‘em up there girls!’ [quote from advertisement].* I was shocked that people found that offensive, genuinely did not see the offense in that whatsoever. (P16)

While censorship of menstruation and dysmenorrhea was largely described in the context of Irish media and education, two students reported that information on menstruation was more difficult to access in their home countries of Malaysia and India than in Ireland.

In discussing formal education, participants indicated that period pain management was virtually unaddressed during primary and secondary education. Some participants were unsure if they had received any information on menstruation, while others recalled receiving a limited amount of information centred on the biological basis of periods. Pain-management and discussion of symptoms were not mentioned as key features of primary education on periods, where these classes were recalled. Moreover, while primary schools in Ireland cater to all sexes and genders and often do so in a mixed environment, a number of participants recalled the practice of removing boys from classrooms during formal talks on menstruation. This early memory reinforced the idea of concealment and was viewed as restricting social discourse around menstruation and period pain.

## Discussion

The objective of the current study was to gather first-hand accounts of dysmenorrhea among university students to identify barriers affecting management and their needs in coping with dysmenorrhea using reflexive TA. Overall, students with dysmenorrhea in this self-selected sample faced debilitating recurrent symptoms of dysmenorrhea and received little guidance on effectively managing menstrual pain during education. Students reported applying heat to their abdominal and/or pelvic regions and using a mix of pharmacological strategies to combat pain, from OTC analgesics to contraception, but were critical of the limited relief and/or adverse effects of medications for dysmenorrhea. Moreover, students preferred coping with dysmenorrhea at home but alluded to a number of barriers impeding home-based management, such as social stigma and perceptions of adverse implications for professional work. Inhibitory factors affecting management also included the perception that there are limited options for pain management, perceived pressure to remain productive while enduring period pain, in addition to schools and households, in some cases, proving unsupportive environments for managing pain. As dysmenorrhea carries substantial implications for general wellbeing, knowledge of effective self-management strategies is critical to ensure students can alleviate painful symptoms that reoccur on a monthly basis.

### Unmet information needs for students with dysmenorrhea

Findings suggest that concrete guidance from authoritative sources on menstrual pain is lacking or inaccessible, leaving individuals to gather pain-management tips by themselves over years of enduring dysmenorrhea. In line with the CSM [24, 25], coherence is the primary domain in need of support at menarche to promote effective self-management. While participants indicated that they had identified methods for managing dysmenorrhea using predominantly heat-based techniques, hormonal contraception, or OTC analgesics, there was notable frustration with the time and effort that was required to construct suitable pain-management routines. The results also indicate that poor menstrual health literacy may function in tandem with social stigma around menstruation, where perceptions of ‘menstrual etiquette’ suppress open communication on periods and dysmenorrhea. In this vein, the findings of Kissling [22], which highlighted that young women face contradictory and undermining social messages regarding menstruation, remain ever-relevant in Ireland over two decades following publication.

In a review of menstrual literacy across low- and high-income countries, Holmes and colleagues [9] indicate that the menstrual information needs of young people fail to be met at any cultural or demographic level. The current study argues that students in Ireland are no exception to this failure, underscoring the importance of providing guidance on coping with dysmenorrhea. Specifically, assumptions cannot be made that individuals who menstruate will have access to menstrual health education prior to menarche and throughout their reproductive years. In Ireland specifically, a recent study by Durand and colleagues [15] notes that students may not have adequate knowledge of evidence-based pain relief strategies to inform their health decisions. As limited menstrual health literacy is likely to affect dysmenorrhea self-management [8, 9], the ‘trial-and-error’ process of identifying effective pain-management strategies may be misguided, misinformed, or may cause young people to endure symptoms of dysmenorrhea without respite.

Moreover, while the current cohort demonstrates a high help-seeking rate compared to similar studies [1, 8], a concerning feature of the data is that consultations with medical professionals did not necessarily facilitate greater understanding of or relief from menstrual pain. Some students felt the need to justify their desire for consultations on menstrual pain, indicating that they felt some doctors may not have regarded their pain as a valid concern. Undermining the severity of menstrual pain by prefixing the qualifier ‘just’ was also commonly reported, where students are faced with an unhelpful label of being *‘just unlucky.’* This finding echoes that of Chen and colleagues [1] whereby individuals with dysmenorrhea are likely to hesitate to seek guidance for pain due to possible dismissal of their concerns by medical professionals or due to internalised views of pain ‘normalcy.’ Additionally, participants perceived that the objective of some consultations was often directed towards eliminating their period entirely. While menstrual suppression is an evidence-based approach for relieving dysmenorrhea [34], some individuals expressed a greater desire to improve their knowledge of active pain management techniques. More comprehensive education on menstrual and gynaecological healthcare for medical professionals as well as shared decision-making opportunities regarding aspects of pain management may improve healthcare experiences for people with dysmenorrhea.

Students with dysmenorrhea in Ireland, therefore, face the challenge of acquiring accurate information on menstrual pain self-management from a social environment that does not facilitate discussion of periods. In addition to poor provision of menstrual health literacy, the current study also indicates that educational spheres may contribute to these prevailing attitudes of menstrual shame. Among students who grew up in Ireland, reports of poor menstrual education in primary school was supplemented with claims that boys were removed from classrooms when menstruation was formally taught, contributing to early perceptions of shame. As young people in Ireland reach menarche at a mean age of 12.5 years [15], inadequate preparation at primary level combined with early censorship is a prominent concern and may contribute to widespread use of ineffective coping strategies for dysmenorrhea [8]. Students registered the effect that such societal practices had on their own experiences, such as feeling unprofessional disclosing to work colleagues that they were experiencing dysmenorrhea. As such, while participants did not view period pain as an inappropriate topic of conversation with friends, their efforts to seek more information on dysmenorrhea were curtailed by prevailing social attitudes ascribing shame and indecency to menstruation as a whole.

### Strength and limitations

In terms of limitations, while attempts to contextualise the experience of students were made in terms of university and cultural background, the current study did not explicitly request participants’ domains of study. Students within healthcare fields may be more comfortable discussing pain and menstruation compared to students in other fields, due to the frequency with which they encounter topics in health and illness. It is also noteworthy that participants demonstrated a considerably high help-seeking rate when compared to similar studies [1, 15]. Views captured here pertain to self-selecting individuals who had a history of help-seeking for dysmenorrhea, a behavioural trend that is not the norm among individuals who menstruate [1, 17]. Furthermore, while none of the participants had known diagnoses, we cannot state with certainty that they did not have any underlying gynaecological conditions that may have contributed to or otherwise affected their pain experiences. Limitations notwithstanding, the current study makes an important contribution through its emphasis on experiential, lived aspects of dysmenorrhea self-management. Strengths of the present study include the rigorous analysis of an understudied yet highly prevalent topic affecting up to 95% of individuals who menstruate [4] and grounding dysmenorrhea within the social context, in light of traditionally negative views of menstruation. Moreover, the present study emphasised the use of inclusive terms during recruitment and throughout data collection, as literature to date is severely lacking data on individuals within the LGBTQ + community who menstruate. Moving forward, a clear effort needs to be made to ensure these voices are represented in menstrual health research.

### Conclusions and recommendations

Limiting access to accurate information on menstrual pain, whether through socially-mediated disincentivisation or omitting concrete information, has wide-ranging effects on individuals who menstruate. Given that recent systematic review evidence suggests dysmenorrhea may be a general risk factor for chronic pelvic and nonpelvic pain [35], there is a clear need to optimise menstrual pain management in adolescence. The current results indicate a need to reform menstrual education, as individuals are not guaranteed an open home environment to learn about menstrual pain. Future research should prioritise the development of a school- or university-based ‘toolkit’ on understanding and managing dysmenorrhea for individuals with menstrual pain to aid individual coherence. Moreover, as many participants expressed relief at COVID-19 ‘Work from Home’ measures, further exploration of infrastructure necessary to facilitate flexible work-from-home or menstrual leave schemes for those with recurrent pain should be emphasised. A potential model for Ireland has recently come into effect in Spain, where new legislation enables individuals with dysmenorrhea to officially avail of three days of menstrual leave per month [36]. As the temporary COVID-19 restrictions granted students in Ireland the opportunity to manage dysmenorrhea from home whilst engaging in their education, the introduction of similar official measures in Ireland as in Spain may facilitate greater dysmenorrhea management. Further exploration of infrastructure necessary to facilitate flexible work-from-home or menstrual leave schemes for those with recurrent pain should be emphasised. Given limited guidance for devising coping strategies for dysmenorrhea, in addition to the unsympathetic social environment for menstrual pain, it appears that students are unsupported and possibly uninformed as they navigate monthly pain. Improving menstrual health literacy and knowledge of available care options for individuals with dysmenorrhea should be classed as a current priority, particularly given the complex relationship between debilitating dysmenorrhea symptoms and health choices for pain management.

## Supplementary Information

**Additional file 1. **COREQ checklist.

**Additional file 2. **Interview topic guide.

## Data Availability

Data supporting the present findings are not publicly accessible due to ethical responsibilities for data protection. Pseudonymised data, however, may be available on reasonable request to the corresponding author.

## Abbreviations

OTC: Over-the-counter
NSAIDs: Non-steroidal anti-inflammatory drugs
CSM: Common sense model of self-regulation
TA: Thematic analysis
COREQ: Consolidated criteria for reporting qualitative research
