# Supplementary figures and images for: Coping with dysmenorrhea: a qualitative analysis of period pain management among students who menstruate
Source: BMC Womens Health. 2022 Oct 5;22:407. doi: 10.1186/s12905-022-01988-4 (PMC9533282; doi:10.1186/s12905-022-01988-4)

**
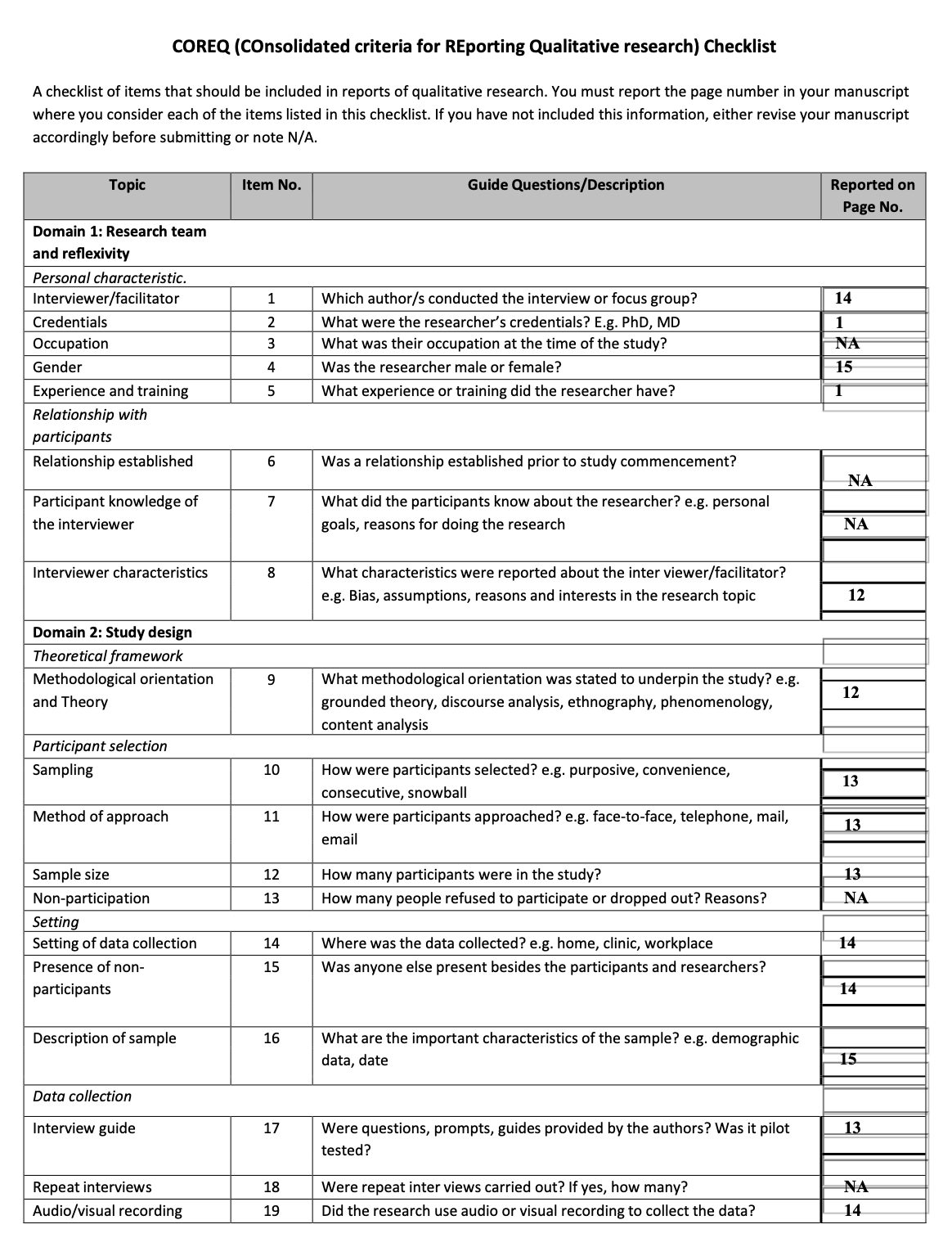
**

**
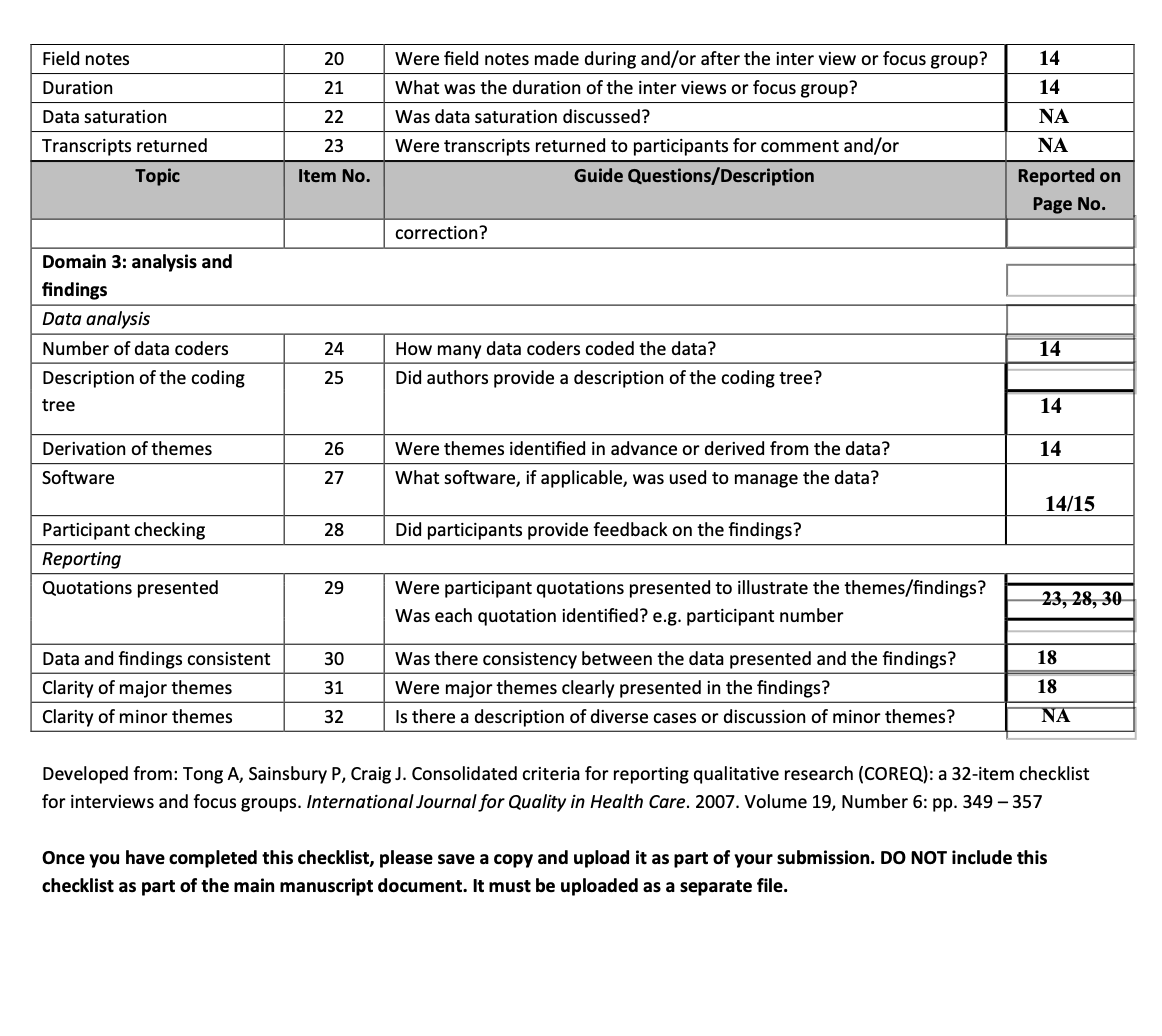
**

Supplement: Supplementary file 1 — Additional file 1. COREQ checklist. [file 12905_2022_1988_MOESM1_ESM.docx]
