# Supplementary material for: Coping with dysmenorrhea: a qualitative analysis of period pain management among students who menstruate
Source: BMC Womens Health. 2022 Oct 5;22:407. doi: 10.1186/s12905-022-01988-4 (PMC9533282; doi:10.1186/s12905-022-01988-4)
Supplement: Supplementary file 2 — Additional file 2. Interview topic guide. [file 12905_2022_1988_MOESM2_ESM.pdf]

	**Interview Topic Guide – Ní Chéileachair et al.**
	**GENERAL EXPERIENCE OF PERIOD PAIN**
	• Can you talk me through what a typical period for you is like?<br>o Probe for symptoms/classification of pain<br>• How would you describe the period cramps in your own words?<br>• In your experience, what are the main problems you face with your menstrual pain?<br>• How equipped do you feel in managing bad period pain?<br>o Probe: What does management involve for you?<br>o Probe: Do you find period pain controllable?<br>• Is there anything you find helpful in coping with pain?<br>o Probe: Is there anything that doesn't work well for you?
	**PSYCHOLOGICAL ASPECTS OF PERIOD PAIN**
	• How do you find your mood during painful periods?<br>o Probe: How does that affect your every-day?<br>• Does your experience with period pain affect your studies?<br>• Has your period ever caused you to miss school/university/work?<br>• Did you ever approach someone or feel you could approach someone about missing school/university/work?<br>o Probe: Why is that? *(if appropriate)*<br>• Do you feel your period in general disrupts any daily activities?<br>o Probe: What about sleep? / hobbies? / daily routine?<br>• What kind of supports do you feel you have available to you?
	**EDUCATION & MENSTRUAL HEALTH LITERACY**
	• How did you learn about aspects of menstrual health, like period pain?<br>o Probe: Is there anything that helped you in learning about period pain?<br>o Probe: Is there anything that blocked or interfered with you in learning about period pain?<br>• Where would you primarily get your information about coping with pain?<br>• In your view, what are the most important pieces of information for coping with period pain?<br>• Are there educational supports available for period pain management?<br>o Probe: What educational supports do you feel individuals who suffer with menstrual pain need in order to cope effectively?

	**SOCIAL FACTORS IN MENSTRUAL PAIN**
	• How comfortable do you feel speaking openly about your experience with period pain?<br>o Probe: Why is that?<br>o Probe: Have you ever been offered help? *(if appropriate)*<br>o Probe: Have you ever thought about approaching a healthcare practitioner or a doctor about it? Why is that?<br>• Thinking specifically about your experience with period pain, do you feel access to healthcare is available for you as a student?<br>o Probe: In your view, what would prevent you from accessing healthcare?<br>• Did you ever feel like you could seek help for the pain?<br>o Probe: Why is that? *(if appropriate)*
	**ISSUES IN LEGITIMACY**
	• In your view, how are menstrual issues approached by the wider public?<br>• How would you describe the attitudes of others towards menstrual pain?<br>o Probe: Do you think people are generally understanding/ considerate?
	**CATCH-ALL NET**
	• From your experience with period pain, is there anything you'd like to mention that we haven't discussed at this point?
